# Diversity and functional roles of endophytic and rhizospheric microorganisms in *Ophioglossum vulgatum* L.: implications for bioactive compound synthesis

**DOI:** 10.3389/fmicb.2025.1618667

**Published:** 2025-07-10

**Authors:** Xian-Nv Long, Xing-Kai Zhang, Yue Wu, Shu-Shuang Tang, Tai-Xiong Zheng, Di Chen, Guan-Hua Cao, Xu-Hong Zhou, Sen He

**Affiliations:** ^1^School of Chinese Materia Medica and Chinese Pharmaceutical Research International Science and Technology Cooperation Base of Yunnan University of Chinese Medicine, Kunming, Yunnan, China; ^2^State Key Laboratory for Quality Ensurance and Sustainable Use of Dao-di Herbs, Beijing, China; ^3^Yunnan Key Laboratory of Southern Medicinal Utilization, Yunnan University of Chinese Medicine, Kunming, China

**Keywords:** plant endophytes, rhizosphere soil, community diversity, bioactive compounds, *Ophioglossum vulgatum*

## Abstract

**Background:**

*Ophioglossum vulgatum* L. is a widely utilized medicinal plant, with the entire plant being used for medicinal purposes. This study systematically characterized the endophytic and rhizospheric community structure, taxonomic diversity, and symbiotic networks within distinct compartments of *O. vulgatum*, while evaluating their potential associations with the accumulation of pharmacologically active metabolites.

**Methods:**

Endophytic and rhizospheric community profiling was conducted via Illumina sequencing, while bioactive compounds were identified using UPLC-ESI-MS/MS.

**Results:**

Roots and leaves harbored beneficial bacteria (e.g., *Methylobacterium*, *Streptomyces*, *Sphingomonas*, and *Flavobacterium*). Dominant fungi included *Archaeorhizomyces* (rhizosphere soil) and *Homophron* (roots/leaves). Dark septate endophytes (DSEs; e.g., *Cladosporium*, *Cladophialophora*, and *Chaetomium*) were abundant across rhizosphere soil, roots, and leaves. Alpha/beta diversity analyses showed higher microbial richness in rhizosphere soil than in plant tissues. Functional predictions (PICRUSt2/FUNGuild) linked endophytic and rhizospheric bacteria to metabolism, human diseases, and biological systems. Network analysis highlighted Basidiomycota as keystone taxa, with modular community structure. Functional predictions revealed that endophytic and rhizospheric microorganisms were associated with critical metabolic pathways, particularly in the biosynthesis of flavonoids and alkaloids (primary bioactive compounds). LEFSe analyses highlighted compartment-specific biomarkers: Acidobacteria, Basidiomycota, and Ascomycota were enriched in distinct zones (rhizosphere, roots, and leaves), with Actinobacteria exhibiting highly significant correlations (*P* < 0.01) with flavonoids, lipids, and quinones, while Acidobacteria, Basidiomycota, and Ascomycota were strongly linked to steroids and tannins (*P* < 0.05).

**Conclusion:**

The diversity and abundance of microbial communities in *O. vulgatum* exhibited tissue-specific and rhizosphere-dependent variations, with distinct patterns strongly correlating to bioactive compound accumulation. Notably, biomarker taxa including Actinobacteria, Acidobacteria, Basidiomycota, and Ascomycota demonstrated robust microbe–metabolite interactions, suggesting their critical regulatory role in biosynthesis pathways. These findings establish endophytic-rhizospheric microbiota as key biosynthetic modulators, proposing innovative approaches for enhancing phytochemical production through targeted microbial community manipulation.

## 1 Introduction

Endophytes are microorganisms that inhabit plant tissues, colonizing partially or completely within the tissues or intercellular spaces of various organs in healthy plants without causing any apparent harm (Vandenkoornhuyse et al., 2015; Zhang et al., 2019). They constitute a vital component of the plant microecosystem. Studies have shown that endophytic microorganisms are abundantly present in roots, stems, leaves, flowers, and fruits of plants, where they play essential roles in plant growth, health and response to biotic stresses (Emami et al., 2019; Matsumoto et al., 2021). Increasing evidences has demonstrated that plant endophytes are an important component of the internal environment of medicinal plants (Pandey et al., 2023; Wu et al., 2021). On one hand, they secrete phytohormones and growth-promoting factors that enhance plant development. On the other hand, they modulate the biosynthese and accumulation of bioactive compounds in medicinal plants. Endophytes can increase the host plant’s active constituents through multiple mechanisms: by synthesizing metabolites identical or structurally analogous to the host’s bioactive compounds, or by biotransforming the plant’s original metabolites into novel derivatives, thereby enriching the diversity and yield of pharmacologically active substances (Li et al., 2022; Ouyang et al., 2024; Yan et al., 2019).

Endophytic bacteria are beneficial microorganisms that establish symbiotic relationships with plants by colonizing their tissues without causing disease. They enhance plant health through multiple mechanisms, including improved nutrient uptake, phytohormone modulation, and promotion of growth, stress tolerance, and overall fitness (Afzal et al., 2019; Taulé et al., 2021). These bacteria exhibit remarkable colonization capacity, inhabiting nearly all plant organs of roots, stems, leaves, flowers, and seeds. Among them, key genera such as *Enterobacter*, *Pseudomonas*, *Burkholderia*, *Bacillus*, and *Streptomyces* play critical roles in plant growth promotion, stress resilience, and biosynthesis of bioactive metabolites (Ek-Ramos et al., 2019; Laranjeira et al., 2022; Taghavi et al., 2010; Vurukonda et al., 2018). Endophytic fungi also colonize the internal tissues of nearly all plant species without causing disease, creating a mutually beneficial relationship where the host plant gains enhanced growth and resilience, while the fungi obtain nutrients and shelter (Baron and Rigobelo, 2022; Zhang J. et al., 2024). Dark Septate Endophytes (DSE) is key symbiotic fungi that significantly enhance plant growth and contribute to ecosystem functionality (Bi et al., 2022). DSE is a group of endophytic fungi commonly found in plant root systems, characterized by melanized septate hyphae and microsclerotia (Santos et al., 2021). They play a crucial role in plant-fungi symbiosis, particularly under stressful conditions like salinity and drought, enhancing plant resilience and adaptation (Akhtar et al., 2022). DSE colonization modulates plant growth by influencing cell metabolism, signaling pathways, and phytohormone biosynthesis, enhancing plant development and stress adaptation (Wu et al., 2020).

The rhizosphere, a highly dynamic zone surrounding plant roots, is enriched with root-derived metabolites that recruit a diverse array of microorganisms, creating a hotspot for microbial activity and interactions. Rhizosphere microorganisms are integral to this ecosystem, playing a vital role in mediating plant–soil interactions (Qu et al., 2020; Wang et al., 2017). This microbial community, which includes bacteria, fungi, archaea, actinomycetes, and other functionally diverse microbes, thrives in the soil adjacent to plant roots (Song et al., 2023). These microorganisms drive essential processes such as soil organic matter decomposition, humus formation, and nutrient transformation, thereby playing a fundamental role in producing high-quality medicinal materials and enhancing the accumulation of secondary metabolites (Jacoby et al., 2017; Noman et al., 2021; Song et al., 2023). Investigating the composition and diversity of rhizosphere microbial communities is essential for unraveling the intricacies of plant growth and stress tolerance. This exploration not only deepens our comprehension of plant–microbe interactions but also paves the way for innovative agricultural practices and sustainable ecosystem management.

*Ophioglossum vulgatum* L. is a small, rare, ancient and perennial fern belonging to the genus *Ophioglossum* and the family Ophioglossaceae. It is known by many folk names, such as Yizhijian, Shexucao, and Maoduncao (Hao et al., 2024; Xu G. et al., 2024). The herb is predominantly distributed across the northern hemisphere, typically thriving in moist mountainous areas, riverbanks, and ditches (Hao et al., 2021). *O. vulgatum* is primarily used medicinally in its entirety, valued for its properties of clearing heat, reducing inflammation, detoxifying, acting as an expectorant, and providing sedative effects (Clericuzio et al., 2014; Hao et al., 2024; Zhu et al., 2025). *O. vulgatum* synthesizes diverse bioactive substances, including flavonoids, sterols, polysaccharides, and alkaloids (e.g., sinoacutine, acutumine, michelalbine, stepharine, sinomerine, and disinomenine), which collectively mediate its pharmacological effects. Flavonoids and alkaloids are the two most important pharmacological substances; however, there are conflicting reports regarding its composition, and its synthesis is influenced by various environmental factors, including endophytes (Xu G. et al., 2024; Yousaf et al., 2024). Studies have revealed that climatic factors, including harvesting period, temperature, hours of sunshine, and rainfall, significantly influence the variations in the content of active ingredients in *O. vulgatum* (Qiu et al., 2024).

Plant–microbe interactions in natural environments are complex, further complicated by microbial abundance and diversity, as well as the heterogeneity of their composition (Sarkar et al., 2022). Notably, current research on endophytic communities in *O. vulgatum* remains limited, with particularly scarce investigations into their functional contributions to bioactive compound biosynthesis. In this study, we addressed this gap by analyzing the microbial diversity and community structure in *O. vulgatum* rhizosphere soils, roots, and leaves, identifying the dominant fungi and bacteria, and establish the relationship between bioactive substances and correlating with probiotic bacteria and fungi. This study lays a foundation for elucidating the diversity and community structure of endophytic and rhizosphere microorganisms, as well as the composition of the main bioactive substances. Furthermore, it explores the correlation between microbial communities and the synthesis and accumulation of bioactive substances. These findings provide both theoretical insights and practical methodologies for cultivating *O. vulgatum* with enhanced yields of bioactive constituents.

## 2 Materials and methods

### 2.1 Materials

*Ophioglossum vulgatum* specimens were collected from Renshi Town (30°47′-31°15′N, 107°42′-108°05′E), Kaijiang County, Dazhou City, Sichuan Province, China. All the collected plant specimens were biennial and taxonomically authenticated by Professor Ronghua Zhao (Yunnan University of Chinese Medicine).

### 2.2 Sample preparation

The fresh roots and leaves of *O. vulgatum* were subjected to a rigorous surface sterilization protocol involving sequential washing with sterile distilled water for 30 s, disinfection in 0.1% NaClO solution for 10 min, treatment with 75% ethanol for 5 min, and final rinsing with sterile distilled water for 3–5 times. After sterilization, the surface moisture was carefully removed using sterile filter paper, and the samples were promptly transferred to pre-sterilized 50 ml centrifuge tubes for subsequent microbial diversity analysis (Beckers et al., 2017). Rhizosphere soil was collected by gently shaking roots to remove loose soil, with residual soil brushed off using a sterile tool (Jiawen et al., 2022). Samples were flash-frozen in liquid nitrogen and sent to Chengdu Ronin Biotechnology Company for endophyte diversity and community composition analysis. The root, leaf, and rhizosphere soil samples (three biological replicates per sample type) were assigned unique identifiers: OrR1-OrR3 for roots, OrL1-OrL3 for leaves, and Rp1-Rp3 for rhizosphere soil. Corresponding bacterial (Eb) and fungal (Ef) communities were separately labeled with triplicate identifiers to maintain consistent identification during experimental analyses.

### 2.3 Bacterial and fungal DNA extraction and PCR amplification

Genomic DNA from the roots and leaves containing endophytic microorganisms was extracted and purified using the Zymo Research BIOMICS DNA Microprep Kit (Zymo Research, USA) following the manufacturer’s protocol. The 16S and ITS rRNA genes of the samples were sequenced on the Illumina platform for bacterial and fungal identification, respectively. PCR amplification was performed using the following primers: 515F (5′-GTGYCAGCMGCCGCGGTAA-3′) and 806R (5′-GGACTACHVGGGTWTCTAAT-3′) for endophytic bacteria, and ITS3 (5′-GATGAAGAACGYAGYRAA-3′) and ITS4 (5′-TCCTCCGCTTATTGATATGC-3′) for endophytic fungi, respectively. The PCR system (50 μl) consisted of 10 × PCR Buffer for KOD-Plus-Neo (5 μl), 2.5 mM dNTPs (5 μl), 25 mM MgSO_4_ (3 μl), forward primer (1.5 μl), reverse primer (1.5 μl), 1 U/μl KOD-Plus-Neo (1 μl), template DNA (2 μl), and ddH_2_O (added to a final volume of 50 μl). The PCR thermocycler conditions were as follows: 94°C for 1 min; 30 cycles of 94°C 20 s, 54°C 30 s, and 72°C 30 s; 72°C for 5 min; and a final hold at 4°C. Each sample was prepared in triplicate, and the PCR products of the same sample were pooled (5 μl) and analyzed by 2% agarose gel electrophoresis. The PCR products were purified using the Zymoclean Gel Recovery Kit (D4008).

### 2.4 Illumina library construction and sequencing

Library construction was performed using the NEBNext Ultra II DNA Library Prep Kit for Illumina (BioLabs Inc., New England). Subsequently, purified amplicons were pooled in equimolar ratios and subjected to paired-end sequencing on an Illumina MiSeq PE250 platform (Illumina, San Diego, CA, USA) following standard protocols provided by Ronin Bio (China).

### 2.5 Data processing and community composition analysis

The clipped double-ended sequences were initially processed as raw tags, which subsequently underwent rigorous quality filtering through QIIME2 to generate high-quality clean tags, and then processed through the Deblur algorithm for chimera removal and generation of amplicon sequence variants (ASVs), resulting in an ASV feature table and feature sequences (Palmer et al., 2025; Mohsen et al., 2019). Subsequently, a species classification dataset for the SILVA database was constructed using a Naïve Bayes classifier and used to annotate the ASV feature sequences with species (Li et al., 2024; Bokulich et al., 2013). Normalized data were used for alpha diversity (Chao1, ACE, Shannon, and Simpson) and beta diversity analyses. Alpha diversity indices were calculated using the Vegan package in R (Faith, 1992). Variance differences among samples were assessed by one-way analysis of variance (ANOVA), with multiple comparisons performed using the agricolae package. The Wilcoxon rank sum test and Principal Coordinate Analysis (PCoA) based on weighted UniFrac distances (GuniFrac package) were used to evaluate sample similarities (Lozupone et al., 2011). Functional predictions for endophytic bacteria and fungi were conducted respectively using PICRUSt2 and FUNGuild.

### 2.6 Determination of bioactive compounds

The bioactive compounds in *O. vulgatum* were identified and quantified using ultra-performance liquid chromatography-electrospray ionization tandem mass spectrometry (UPLC-ESI-MS/MS; SCIEX, Shanghai, China). Prior to analysis, the freeze-dried samples from each experimental group were homogenized using a Retsch MM 400 mixer mill (Haan, Germany) operating at 30 Hz for 90 s to ensure complete powderization and sample uniformity for accurate bioactive compound determination. A total of 50 mg of powder was weighed and extracted with 1,200 μl of 70% aqueous methanol, prechilled to −20°C and containing internal standards. The extraction process involved vortexing every 30 min for a total of 6 times, with each vortexing lasting 30 s. The mixture was then centrifuged at 12,000 rpm for 3 min, and the supernatants were filtered through a 0.22 μm filter membrane. The filtrate was stored in a sample vial for subsequent UPLC-ESI-MS/MS analysis (Zhang X. K. et al., 2024).

### 2.7 Statistical analysis

Alpha diversity indices were calculated with the Vegan package in R (Version 4.2.2). Beta diversity was assessed through PCoA based on Bray–Curtis distances, performed using the microeco R package. Venn diagrams were generated with the VennDiagram package in R (Version 4.2.2). Differential species analysis was conducted using the Linear Discriminant Analysis Effect Size (LEfSe) software (Version 1.0). For microbial co-occurrence network construction, we identified representative ASVs that were: (1) consistently detected across all samples, (2) maintained a mean relative abundance ≥0.1% within each experimental group, and (3) demonstrated significant ecological associations (Spearman’s |*r*| > 0.7, *P* < 0.05) through correlation analysis. The co-occurrence network was meticulously constructed using the igraph package in R (Version 4.2.2), capturing the intricate relationships among microbial communities. For enhanced clarity and visual appeal, the network was further refined and visualized using Gephi (Version 0.9.2). To evaluate the potential ecological relationships, Mantel tests were systematically employed to examine the multivariate correlations between: (1) bioactive compounds, and (2) the community structures of both probiotic bacteria and fungi, with statistical significance assessed through permutations.

## 3 Results

### 3.1 Sequencing data statistics and ASV analysis

**FIGURE 1:**
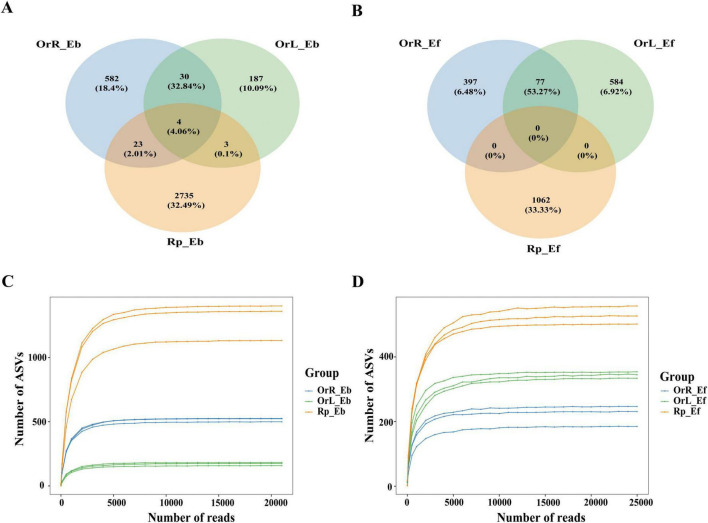
Comparative analysis of endophytic microbial communities in plant compartments and rhizosphere soil. **(A)** Venn diagram of shared bacterial ASVs among rhizosphere soil, root, and leaf samples. **(B)** Venn diagram of shared fungal ASVs. **(C)** Rarefaction curves for endophytic bacterial communities. **(D)** Rarefaction curves for endophytic fungal communities. Rhizosphere soil exhibited the highest ASV richness for both bacteria and fungi, with root-leaf overlaps comprising 30 bacterial and 77 fungal ASVs (>30% of total sequences; fungal overlap reached 50%). Rarefaction analysis confirmed sufficient sequencing depth and reinforced the rhizosphere’s dominance in microbial diversity.

After quality filtering, we obtained 303,817 high-quality bacterial sequences and 312,052 fungal sequences. Following Deblur analysis to eliminate chimeric and somatic sequences, 303,639 high-quality bacterial sequences (mean length: 292 bp) and 311,828 fungal sequences (mean length: 350 bp) (Supplementary Table 1) were retained. Using a 97% similarity threshold, these sequences clustered into 3,564 bacterial ASVs and 2,120 fungal ASVs. Taxonomic classification revealed 35 bacterial phyla (665 genera and 779 species) and 13 fungal phyla (313 genera and 389 species). Venn diagram analysis revealed that bacterial and fungal endophytes were abundant in rhizosphere soil, root, and leaf samples, though species richness differed markedly among these compartments. Rhizosphere soil bacterial communities shared only four ASVs with endophytic bacteria. The rhizosphere exhibited the highest number of unique bacterial ASVs (2,735), compared to roots (582 ASVs), and leaves (187 ASVs). While 23 ASVs were shared between rhizosphere soil and roots, only 3 ASVs overlapped between rhizosphere soil and leaves. Notably, roots and leaves shared 30 bacterial ASVs (Figure 1A). Similarly, endophytic fungal ASVs were significantly more abundant in rhizosphere soil than in root and leaf samples, with 77 ASVs shared between root and leaf endophytic fungal species (Figure 1B). Notably, the total number of endophytic fungal ASVs exceeded that of endophytic bacteria in both leaves and roots. Interestingly, for both bacteria and fungi, shared ASVs between roots and leaves represented > 30% of total sequences, with this proportion reaching 50% for fungi, indicating substantial microbiota overlap between these compartments (Figures 1A, B). Rarefaction curves confirmed adequate sequencing coverage, with rhizosphere soils harboring the highest ASV richness for both bacteria and fungi at equivalent sequencing depths (Figures 1C, D).

### 3.2 Compartment-specific assembly of microbial communities in the plant-soil system

**FIGURE 2:**
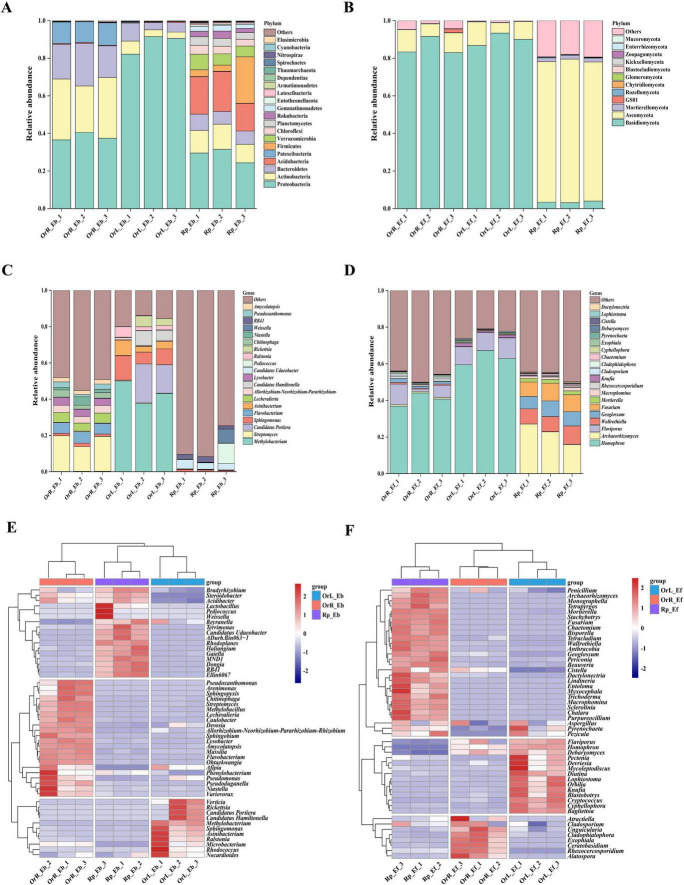
Microbial community composition and structure across *O. vulgatum* compartments. Bacterial/fungal composition at phylum **(A,B)** and genus **(C,D)** levels. **(E,F)** Top 50 genera heatmaps. Proteobacteria dominated all bacterial niches (28.5%–88.1%), contrasting fungal compartmentalization: Ascomycota in rhizosphere (75.0%) vs. Basidiomycota in roots (86.0%)/leaves (90.1%). Key specialists: *Candidatus Udaeobacter* (rhizosphere, 4.0%), *Streptomyces* (roots, 17.5%), and *Methylobacterium* (leaves, 43.5%); *Archaeorhizomyces* (rhizosphere, 21.9%) and *Homophron* (roots/leaves, 40.6%–63.3%). Heatmaps indicate bacterial rhizosphere-leaf convergence vs. fungal root-leaf clustering, suggesting cross-niche microbial transfer.

The composition and assembly of endophytic microbial communities exhibited distinct compartment-specific patterns across the plant-soil system (Figure 2). At the phylum level, bacterial communities in rhizosphere soil were dominated by Proteobacteria (28.48%), Acidobacteria (18.58%), and Actinobacteria (11.73%), while root endophytes showed higher proportions of Proteobacteria (38.17%) and Actinobacteria (29.79%). Leaf bacterial communities were overwhelmingly dominated by Proteobacteria (88.09%), demonstrating this phylum’s remarkable adaptability across compartments (Figure 2A). Fungal communities displayed even stronger differentiation, with Ascomycota dominating rhizosphere soils (75.04%) vs. Basidiomycota predominating in roots (86.01%) and leaves (90.08%) (Figure 2B).

Genus-level analysis revealed further specialization, with *Candidatus Udaeobacter* (4.03%) being most abundant in rhizosphere soil, *Streptomyces* (17.48%) in roots, and *Methylobacterium* (43.51%) in leaves (Figure 2C). Fungal communities showed similar compartment partitioning, dominated by *Archaeorhizomyces* (21.87%) in rhizosphere soil and *Homophron* (40.56%–63.25%) in plant tissues (Figure 2D). Heatmap analysis of the top 50 genera revealed interesting structural patterns: bacterial communities showed similarity between rhizosphere soil and leaves, while fungal communities clustered between roots and leaves (Figures 2E, F).

### 3.3 Differential indicator species analysis

**FIGURE 3:**
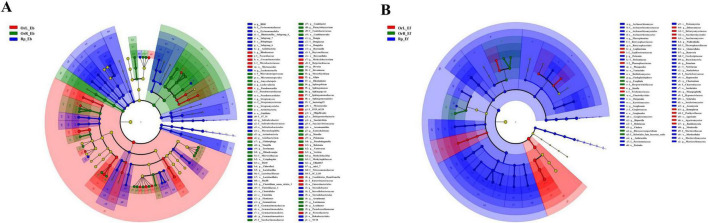
Compartment-specific microbial enrichment patterns revealed by LEfSe analysis. **(A)** Bacterial and **(B)** fungal community differences among *O. vulgatum* rhizosphere soil, roots, and leaves. The cladograms (phylum to genus level) depict taxonomic units with node sizes proportional to relative abundance. Colored nodes represent significantly enriched taxa per compartment (yellow: non-significant). Letters denote key discriminative taxa. Rhizosphere soil contained the highest indicator species counts (54 bacterial and 49 fungal), followed by roots (36 bacterial and 6 fungal), and leaves (22 bacterial and 14 fungal), indicating pronounced compartment-specific selection in *O. vulgatum*-microbe interactions.

Linear Discriminant Analysis Effect Size analysis revealed distinct microbial taxa associated with different compartments of *O. vulgatum*, identifying 112 bacterial and 69 fungal lineages with significant compartment preferences (Figure 3). The rhizosphere soil exhibited the highest microbial specificity, containing 54 bacterial and 49 fungal indicator species, including Acidobacteria, Firmicutes, and Gemmatimonadetes (represented by *Succinivibrio* and *Steroidobacter*) among bacteria (Figure 3A), and Ascomycota (particularly *Archaeorhizomyces* and *Fusarium*) among fungi (Figure 3B). In contrast, root endophytes were dominated by Actinobacteria (e.g., *Amycolatopsis* and *Streptomyces*) (Figure 3A) and specialized fungi (Figure 3B) such as *Cladophialophora* and *Exophiala*, with 36 bacterial and only 6 fungal indicators detected. Leaf tissues showed a different pattern, enriched with Proteobacteria (*Methylobacterium* and *Sphingomonas*) (Figure 3A) and Basidiomycota fungi (*Homophron* and *Debaryomyces*) (Figure 3B), comprising 22 bacterial and 14 fungal indicator species.

### 3.4 Analysis on the microbial diversity

**FIGURE 4:**
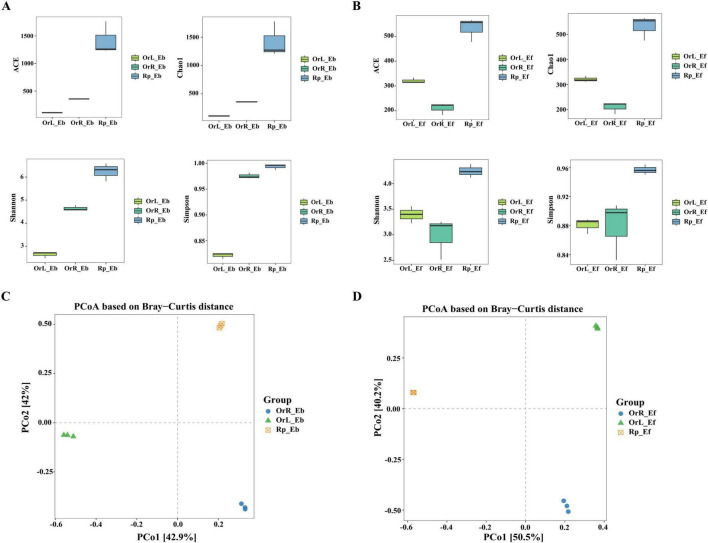
Microbial diversity patterns across *O. vulgatum* compartments. Alpha diversity of **(A)** bacterial and **(B)** fungal communities showing significantly higher richness (Chao1) and diversity (Shannon/Simpson) in rhizosphere soil vs. roots/leaves (*P* < 0.05, ANOVA). PCoA plots of **(C)** bacterial (84.9% variance explained) and **(D)** fungal (90.7% variance) communities based on Bray–Curtis distances, demonstrating clear compartment differentiation.

Alpha and beta diversity analyses revealed distinct microbial community patterns across *O. vulgatum* compartments (Figure 4). Bacterial richness (Chao1/ACE indices) and diversity (Shannon/Simpson) showed a consistent soil > root > leaf gradient (*P* < 0.05), with rhizosphere communities exhibiting 1.5–2-fold higher values than plant tissues (Figure 4A). In contrast, fungal diversity followed a soil > leaf > root pattern, suggesting stronger tissue-specific adaptation in fungi (Figure 4B). Beta diversity analysis (PCoA/Bray–Curtis) confirmed these trends, with clear separation of bacterial (84.9% variance explained) and fungal (90.7% variance) communities among compartments (Figures 4C, D). Notably, despite physical proximity, rhizosphere and root communities formed distinct clusters, indicating independent assembly mechanisms rather than passive microbial migration. These findings demonstrate that the endophytic bacterial and fungal community compositions in rhizosphere soil, roots, and leaves are markedly distinct. Despite prolonged contact between roots and rhizosphere soils, their separation in the PCoA analysis suggests that the sources of endophytic bacterial and fungal communities in these compartments are not the same.

### 3.5 Functional potential of endophytic communities

**FIGURE 5:**
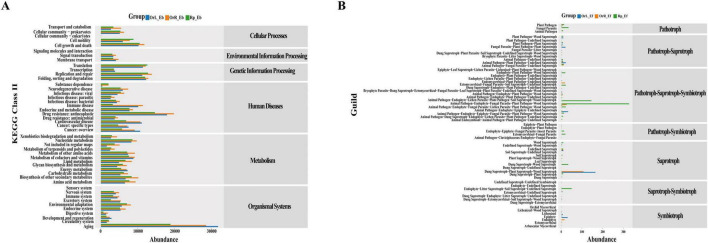
Functional profiling of endophytic communities in *O. vulgatum* and rhizosphere soil. **(A)** PICRUSt2 analysis revealed that genes associated with human diseases, metabolism, and organismal systems exhibited high relative abundance. A detailed predictive classification of ecological functions identified a total of 46 metabolic pathways across the samples. **(B)** FUNGuild analysis showed that Saprotroph and Pathotroph-Saprotroph-Symbiotroph functional groups dominated in rhizosphere soil, roots, and leaves. Additionally, a detailed predictive categorization of species ecological functions highlighted distinct distributions of dominant functional taxa across different compartments.

Functional prediction analyses revealed distinct metabolic and ecological stratification of endophytic communities across *O. vulgatum* compartments (Figure 5). PICRUSt2 analysis identified six major KEGG pathways in bacterial communities, with human disease-related, metabolic, and organismal system pathways showing the highest relative abundance. Notably, 46 metabolic sub-pathways were detected, including root-enriched pathways involved in aging, antineoplastic drug resistance and nucleotide metabolism, as well as rhizosphere-abundant pathways related to cell growth/death and immune diseases (Figure 5A). Fungal functional guild analysis demonstrated clear compartment partitioning: rhizosphere soil was dominated by multifunctional Pathotroph-Saprotroph-Symbiotrophs (68.4%) involved in complex nutrient cycling and plant–microbe interactions, while roots harbored primarily Saprotrophs (53.7%) alongside symbiotic and pathogenic fungi. Leaves maintained a balanced Saprotroph consortium (53.6%) with significant representation of both decomposer and pathogenic functions (Figure 5B). These functional patterns suggest that *O. vulgatum* selectively enriches microbiota with complementary metabolic capabilities-bacterial communities in roots appear specialized for metabolic processing and stress response, while rhizosphere fungi exhibit broader ecosystem-engineering capacities through diverse trophic strategies. Such compartmentalized functional specialization likely underlies the plant’s ability to maintain beneficial microbial partnerships while optimizing resource acquisition and defense across different tissues.

### 3.6 Analysis of co-occurrence network

**FIGURE 6:**
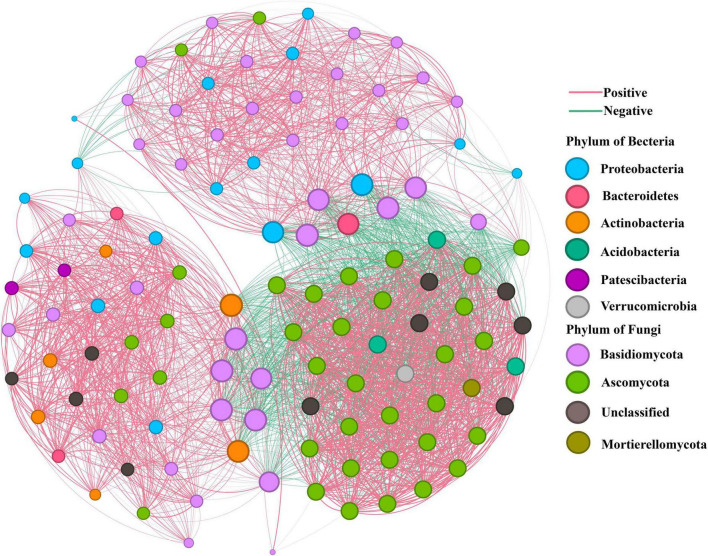
Bacterial-fungal co-occurrence network in *O. vulgatum* ecosystems. The network visualization displays ASVs (97% similarity threshold) as nodes colored by phylum affiliation, with node size proportional to connectivity degree. Significant correlations (*P* < 0.05, |*r*| > 0.70) are shown as edges (positive: pink; negative: green). Key topological features include: (1) dominance of Basidiomycota (largest nodes) as keystone taxa, (2) network complexity (116 nodes, 2,585 edges; average degree = 44.6), and (3) structural robustness (modularity = 0.441 > 0.40 threshold; clustering coefficient = 0.875). The 3.2:1 ratio of positive:negative edges (74.8% vs. 25.2%) highlights predominantly mutualistic microbial interactions across rhizosphere, roots, and leaves. Graph density (0.388) further confirms tight ecological connectivity within this microbiome system.

Co-occurrence network analysis unveiled the intricate web of interactions between bacterial and fungal communities within the rhizosphere soil, roots, and leaves of *O. vulgatum* (Figure 6). In the *O. vulgatum* network, the majority of nodes were affiliated with six dominant bacterial phyla and four dominant fungal phyla. These were primarily composed of Basidiomycota, Ascomycota, Proteobacteria, Actinobacteria, and Bacteroidetes, with Basidiomycota exhibiting a significantly higher relative abundance compared to the others. The network comprised 116 nodes and 2,585 edges, where positive correlations signified cooperative interactions, while negative correlations reflected competition with the host or inhibitory relationships among microorganisms. Notably, the proportion of positive correlations among rhizosphere soils, roots, and leaves (74.82%) surpassed that of negative correlations (25.18%), underscoring a strong tendency toward microbial cooperation. Network analysis further revealed that the modularity index of the bacterial-fungal network was 0.441, exceeding the threshold of 0.40, which suggests the presence of a distinct modular structure within the *O. vulgatum* community. The network displayed high complexity (average degree = 44.6, graph density = 0.388) and strong modular organization (modularity index = 0.441), suggesting compartment-based functional specialization among microbial groups. Furthermore, the exceptionally high clustering coefficient (0.875) demonstrates remarkable structural stability, implying resilience to environmental perturbations. These network properties collectively suggest that *O. vulgatum* maintains a highly interconnected microbial consortium where cooperative interactions prevail, particularly involving Basidiomycota fungi as potential keystone taxa. Such stable, mutualism-dominated network architecture likely enhances the plant’s ability to recruit beneficial microbiota while buffering against external stresses, ultimately contributing to host fitness and ecosystem functioning.

### 3.7 Correlation between microbial communities and bioactive compounds

**FIGURE 7:**
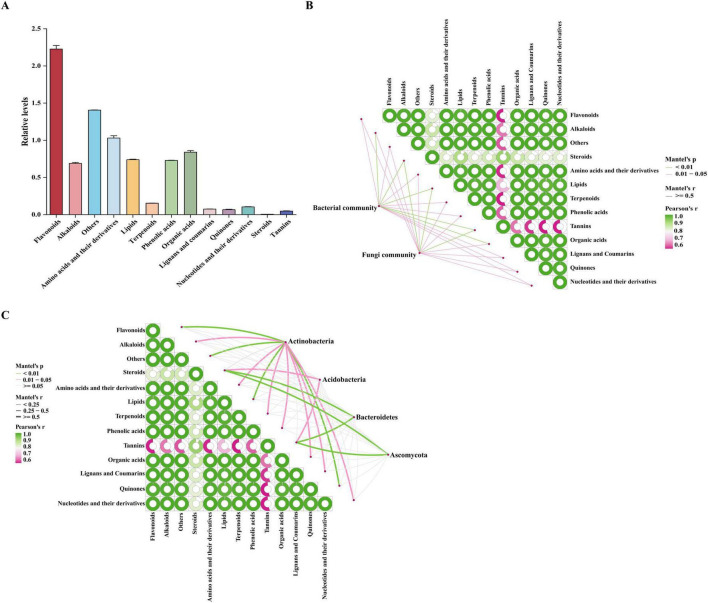
Bioactive compound profiles and microbe-metabolite correlations in *O. vulgatum*. **(A)** Relative abundance of 2,261 identified metabolites (normalized values × 108), showing flavonoids as the dominant class followed by amino acid derivatives and organic acids. **(B)** Mantel test results demonstrating significant positive correlations between microbial communities and key metabolites: bacterial associations with alkaloids, steroids and lipids (*P* < 0.01), and fungal links to flavonoids and phenolic acids (*P* < 0.05). **(C)** Actinobacteria exhibited highly significant positive correlations with flavonoids, lipids, and quinones (*P* < 0.01), whereas Acidobacteria along with Basidiomycota and Ascomycota fungi showed significant associations with steroids and tannins (*P* < 0.05). Significant interactions were observed among the bioactive compounds themselves, revealing potential co-regulation within metabolic networks.

This study characterized the relationship between microbial communities and bioactive compound production in *O. vulgatum* through comprehensive metabolite profiling and correlation analysis. We identified 2,261 secondary metabolites, with flavonoids (583 compounds), amino acid derivatives (246), and organic acids (104) representing the most abundant classes, followed by lipids (262), alkaloids (183), and phenolic acids (190) (Supplementary Table 2 and Figure 7A). Mantel tests revealed significant microbe-metabolite correlations (*P* < 0.05): both bacterial and fungal communities showed strong positive associations with steroids, lipids, tannins, flavonoids, amino acid derivatives, terpenoids, and phenolic acids, while bacteria additionally correlated (*P* < 0.01) with alkaloids and organic acids (Figure 7B). Further analysis revealed that phylum-level biomarker taxa more distinctly emphasized specific interactions: Actinobacteria exhibited strong positive correlations (*P* < 0.01) with flavonoids, lipids, and quinones, whereas Acidobacteria along with the fungal phyla Basidiomycota and Ascomycota showed significant correlation (*P* < 0.05) with steroids and tannins (Figure 7C). In addition, we observed extensive positive correlations among the metabolites themselves, particularly between flavonoids, alkaloids, and amino acid derivatives, suggesting tightly coordinated biosynthetic pathways. These findings provide compelling evidence that *O. vulgatum*’s microbial communities play a crucial role in regulating the production of its valuable bioactive compounds, likely through direct synthesis or modulation of plant metabolic pathways.

## 4 Discussion

Plant endophytic and rhizospheric microorganisms play pivotal roles in mediating host growth and secondary metabolite synthesis, with profound implications for both natural ecosystems and commercial applications (Li et al., 2023). Our study reveals that *O. vulgatum* harbors a remarkably diverse endophytic and rhizospheric microbiome, comprising 3,564 bacterial ASVs (spanning 35 phyla) and 2,120 fungal ASVs (13 phyla), exhibiting distinct compartment partitioning across rhizosphere soil, roots, and leaves (Figure 1). The observed microbial richness gradient, with rhizosphere soils as the most biodiverse compartment, followed by roots and leaves for bacteria, and leaves surpassing roots for fungi that aligns with established ecological theory predicting stronger environmental filtering in plant interior tissues. This pattern likely reflects the combined effects of (1) soil as a microbial source habitat, (2) root exudate-mediated selection, and (3) leaf-specific physicochemical constraints (Dang et al., 2023).

The predominance of Proteobacteria, Actinobacteria, and Bacteroidetes among bacterial communities mirrors findings in other medicinal plants like *Lonicera japonica* (Xu Q. et al., 2024) and *Pinus bungeana* (Yang et al., 2022), suggesting conserved phylum-level associations across phylogenetically distant hosts (Figure 2A). Notably, the functional specialization of these taxa, including nitrogen fixation (Proteobacteria), bioactive metabolite production (Actinobacteria), and organic matter decomposition (Bacteroidetes), considering them as key players in potentially enhancing *O. vulgatum*’s medicinal properties through direct synthesis or stimulation of plant metabolic pathways (Narsing et al., 2022; Pan et al., 2023). The fungal community’s distinct distribution pattern (rhizosphere > leaves > roots), contrasting with bacterial trends, may reflect the difference in the roles of active compound biosynthesis or compartment-specific resource availability, warranting further investigation into fungal-plant biochemical crosstalk (Figure 4B).

The predominance of Ascomycota and Basidiomycota across all examined compartments of *O. vulgatum* aligns with observations in other plant systems such as *Stipa purpurea* and *Picea abies* (Marčiulynas et al., 2022; Yang et al., 2020), suggesting conserved functional roles of these phyla in plant–microbe ecosystems (Figure 2B). Notably, their compartment-specific distribution patterns reveal distinct ecological strategies: Ascomycota demonstrated particularly high abundance in rhizosphere soils, consistent with their well-documented saprophytic capabilities in decomposing recalcitrant organic compounds (Beimforde et al., 2014; Gaiero et al., 2013). In contrast, Basidiomycota showed pronounced dominance in roots and leaves, likely reflecting their dual ecological roles as both decomposers and symbiotic partners. Their prevalence in plant tissues may be attributed to their unique enzymatic machinery for breaking down complex plant polymers like lignin and cellulose (Manici et al., 2024), as well as their ability to form mycorrhizal associations that enhance host nutrient acquisition (Frey et al., 2004). This compartment partitioning between Ascomycota (soil specialists) and Basidiomycota (plant tissue specialists) suggests complementary functional contributions to plant health—while Ascomycota maintain soil nutrient pools through organic matter decomposition, Basidiomycota directly facilitate nutrient transfer to the host plant. The conservation of these phylum-level associations across diverse plant species, including our findings in *O. vulgatum*, highlights their fundamental importance in plant–microbe systems. Network analysis also identified Basidiomycota as the keystone microbial group in *O. vulgatum*, exhibiting the highest relative abundance among four fungal and six bacterial phyla (Figure 6). Its central network position and numerous positive correlations highlight its critical role in maintaining this stable, cooperative microbiome, likely through organic matter decomposition, nutrient cycling functions and active compound biosynthesis (Banerjee et al., 2018).

Our genus-level analysis revealed distinct compartmentalization of microbial communities in *O. vulgatum*, with each compartment harboring specialized taxa exhibiting unique functional attributes (Figure 2). The rhizosphere was dominated by *Candidatus Udaeobacter*, a trace gas-scavenging bacterium known to maintain soil microbial equilibrium through antibiotic production (Willms et al., 2020). Root endophytes were characterized by *Streptomyces*, the prolific producers of bioactive metabolites that account for approximately 65% of known microbial-derived plant growth regulators (Law et al., 2018; Pang et al., 2022). This genus’s prevalence suggests its potential role in mediating *O. vulgatum*’s secondary metabolism and stress resistance. The leaf microbiome showed strong selection for *Methylobacterium*, nitrogen-fixing symbionts that enhance host disease resistance through phytohormone modulation (Sanjenbam et al., 2022). The consistent presence of plant growth-promoting bacteria (PGPB) across compartments, including nitrogen-fixing *Allorhizobium* groups, phosphorus-solubilizing *Pseudoxanthomonas*, and siderophore-producing *Sphingomonas* (Singh et al., 2022), demonstrates *O. vulgatum*’s ability to cultivate beneficial consortia throughout its tissues. Fungal communities exhibited parallel compartment specialization, with the rhizosphere-enriched *Archaeorhizomyces* enhancing mineral nutrient acquisition (Ma et al., 2022), while the root/leaf-associated *Homophron* (Basidiomycota) conferred stress resilience (Rosas-Medina et al., 2020). The widespread colonization by DSEs like *Cladophialophora* and *Exophiala* suggests their potential involvement in both nutrient mobilization and bioactive compound synthesis (Wang et al., 2022). LEfSe analysis confirmed strong compartment differentiation, identifying 112 bacterial and 69 fungal biomarkers. Acidobacteria, Basidiomycota, and Ascomycota were distinctly enriched in *O. vulgatum*’s rhizosphere soil, roots, and leaves, respectively (Figure 3). This compartmentalization parallels patterns seen in *Cycas panzhihuaensis* (Zheng and Gong, 2019), suggesting conserved evolutionary strategies for microbiome partitioning across phylogenetically distant plants. The consistent presence of PGPB in all compartments implies that *O. vulgatum* actively maintains beneficial microbial functions throughout its tissues, potentially contributing to its medicinal properties.

Our study reveals a sophisticated compartmentalization of microbial communities in *O. vulgatum*, with distinct diversity patterns and functional specialization across different plant tissues and rhizospheric soil. The alpha diversity analysis demonstrated that bacterial communities followed a clear rhizosphere soil > roots > leaves gradient, while fungal communities exhibited a modified pattern of rhizosphere soil > leaves > roots (Figure 4). These findings align with previous observations in *Senecio vulgaris* L. (Cheng et al., 2019) and support the emerging paradigm of plant-mediated microbiome assembly across phylogenetically diverse species (Trivedi et al., 2020). Beta diversity analysis further confirmed significant compositional differences between compartments (*P* < 0.01), underscoring the strong selective pressures exerted by plant tissues through their unique physicochemical microenvironments (Zhang et al., 2023). The functional predictions provided deeper insights into potential microbe–host interactions, revealing that bacterial metabolic profiles were particularly enriched in human disease-related pathways and metabolic processes (Figure 5A), potentially linked to *O. vulgatum*’s characteristic accumulation of flavonoids and alkaloids. Fungal communities displayed particularly interesting compartment specialization patterns, with the rhizosphere dominated by multifunctional Pathotroph-Saprotroph-Symbiotrophs, reflecting the complex nutrient dynamics of soil ecosystems (Figure 5B). In contrast, roots and leaves were enriched in Saprotrophs, likely supporting organic matter decomposition in these tissues. This guild distribution mirrors patterns observed in agricultural systems (Clocchiatti et al., 2020), where saprotrophs are known to enhance soil stability through their extensive hyphal networks (Abiven et al., 2007). The dynamic equilibrium between different fungal nutritional modes appears particularly significant for plant health, where symbiotroph dominance enhances host resistance while potentially suppressing saprotrophic activity, and pathotroph prevalence correlates with disease susceptibility.

These findings collectively suggest that *O. vulgatum* has evolved to maintain compartmentalized microbial functions that likely contribute to its medicinal properties through several mechanisms: the rhizosphere microbiome facilitates nutrient mobilization, root endophytes may participate in secondary metabolite production, and leaf-associated microbes could influence defense compound synthesis. Through comprehensive metabolite profiling, we identified 2,261 secondary metabolites, with flavonoids (583 compounds), amino acids and derivatives (246 compounds), and organic acids (104 compounds) representing the most abundant classes (Figure 7A and Supplementary Table 2). These results align with previous reports on the chemical composition of *O. vulgatum* (Yousaf et al., 2024), confirming its rich pharmacologically active constituents. Mantel test analysis revealed significant microbe-metabolite associations, with bacterial communities showing particularly strong correlations (*P* < 0.01) with alkaloids, steroids, and lipids, while fungal communities were more closely associated (*P* < 0.05) with flavonoids, terpenoids, and phenolic acids (Figure 7B). The strong flavonoid-microbiome correlation may reflect their dual roles in plant defense and microbial signaling (Zeng et al., 2022), while the alkaloid-bacteria associations suggest specialized interactions related to nitrogen metabolism (Fairbairn and Paterson, 1966). These patterns mirror observations in other medicinal plants such as *Panax notoginseng* (Burkill) F. H. Chen ex C. H. Chow and *Paris polyphylla* var. *yunnanensis*, supporting the emerging understanding that endophytic and rhizospheric microorganisms play vital roles in the biosynthesis and accumulation of active compounds (Dai et al., 2024; Shu et al., 2025; Zhang X. K. et al., 2024). In addition, significant correlations were identified between microbial taxa and specialized metabolites: Actinobacteria exhibited a highly significant positive association (*P* < 0.01) with flavonoids, lipids, and quinones, while Acidobacteria, Basidiomycota, and Ascomycota showed marked positive correlations (*P* < 0.05) with steroids and tannins (Figure 7C). These findings that align with previous research demonstrating the critical role of Actinomycetes in flavonoid biosynthesis in *Dendrobium* species (Yuan et al., 2024).

The distribution of microbial functions suggests a coordinated system where: (1) rhizosphere communities facilitate nutrient mobilization and provide precursor molecules, (2) root endophytes (particularly *Streptomyces*) likely participate in alkaloid biosynthesis and nitrogen metabolism, and (3) leaf-associated microbes (notably *Methylobacterium*) may influence flavonoid synthesis and plant defense responses. These compartmentalized interactions appear to have co-evolved to enhance *O. vulgatum*’s medicinal properties through both direct microbial biosynthesis and microbiome-induced plant metabolic pathways.

Taken together, these findings provide valuable insights into the ecological roles of secondary metabolites and establish a foundation for future research to: (1) isolate and characterize key microbial taxa responsible for metabolite production, (2) elucidate the molecular mechanisms of microbe-induced metabolite synthesis, and (3) develop microbiome-based cultivation strategies to enhance medicinal compound yields in *O. vulgatum* and related species.

## 5 Conclusion

This study elucidates the diversity and functional specialization of endophytic and rhizospheric microorganisms in *O. vulgatum* and their pivotal role in bioactive compound synthesis. High-throughput sequencing revealed distinct microbial communities across plant compartments, with Proteobacteria, Actinobacteria, and Bacteroidetes dominating bacterial assemblages, while Ascomycota and Basidiomycota prevailed among fungi. Basidiomycota emerged as a keystone taxon, maintaining network stability. Functional predictions linked endophytic and rhizospheric microorganisms to critical metabolic pathways, particularly in flavonoid and alkaloid biosynthesis, supported by strong microbe-metabolite correlations. Notably, biomarker taxa including Actinobacteria, Acidobacteria, Basidiomycota, and Ascomycota demonstrated robust microbe-metabolite interactions, suggesting their critical regulatory role in biosynthesis pathways. These findings demonstrate how compartmentalized microbial functions, from rhizosphere nutrient mobilization to root/leaf metabolite production, collectively enhance bioactive compound accumulation. This integrated microbiome-metabolome framework provides novel insights for harnessing plant–microbe interactions to optimize medicinal yields in *O. vulgatum* and related species.

## Data Availability

The original contributions presented in the study are publicly available. This data can be found here: https://www.ncbi.nlm.nih.gov, accession number: PRJNA1250425.
