# Supplementary material for: Diversity and functional roles of endophytic and rhizospheric microorganisms in Ophioglossum vulgatum L.: implications for bioactive compound synthesis
Source: Front Microbiol. 2025 Jul 10;16:1618667. doi: 10.3389/fmicb.2025.1618667 (PMC12288683; doi:10.3389/fmicb.2025.1618667)
Supplement: Supplementary file 1 [file Table_1.docx]

**Supplementary Table 1.** Sequencing statistics of endophytic microbial communities across *Ophioglossum vulgatum* L. compartments

| **Endophyte category** | **Sample ID** | **Raw_tags** | **Clean_tags** | **Clean_avg_len** | **Q30 (%)** | **Efficiency_rate (%)** |
| --- | --- | --- | --- | --- | --- | --- |
| Bacteria | OrR_Eb_1 | 34456 | 34433 | 293.8 | 97.69 | 99.93 |
|  | OrR_Eb_2 | 33302 | 33289 | 293.5 | 97.9 | 99.96 |
|  | OrR_Eb_3 | 33991 | 33969 | 294.2 | 97.76 | 99.94 |
|  | OrL_Eb_1 | 32740 | 32716 | 291.1 | 97.94 | 99.93 |
|  | OrL_Eb_2 | 31839 | 31829 | 290 | 97.81 | 99.97 |
|  | OrL_Eb_3 | 31500 | 31479 | 291.3 | 97.93 | 99.93 |
|  | Rp_Eb_1 | 37271 | 37231 | 290.9 | 98.05 | 99.89 |
|  | Rp_Eb_2 | 33940 | 33938 | 291.8 | 98.26 | 99.99 |
|  | Rp_Eb_3 | 34778 | 34755 | 290.8 | 98.01 | 99.93 |
|  | Total | 303817 | 303639 | 292 | 98 | 100 |
| Fungi | OrR_Ef_1 | 33099 | 33078 | 363.6 | 96.27 | 99.94 |
|  | OrR_Ef_2 | 37119 | 37094 | 357.5 | 96.52 | 99.93 |
|  | OrR_Ef_3 | 33789 | 33765 | 359.9 | 96.31 | 99.93 |
|  | OrL_Ef_1 | 34050 | 34007 | 345.2 | 96.46 | 99.87 |
|  | OrL_Ef_2 | 34012 | 33954 | 341.6 | 96.67 | 99.83 |
|  | OrL_Ef_3 | 34200 | 34166 | 343.9 | 96.67 | 99.9 |
|  | Rp_Ef_1 | 33520 | 33507 | 342.2 | 97.38 | 99.96 |
|  | Rp_Ef_2 | 39403 | 39398 | 345.7 | 97.31 | 99.99 |
|  | Rp_Ef_3 | 32860 | 32859 | 346.7 | 97.36 | 100 |
|  | Total | 312052 | 311828 | 350 | 97 | 100 |
